# Evaluation of mechanical properties of different polyetheretherketone endodontic post systems: an in vitro study

**DOI:** 10.1186/s12903-023-03193-7

**Published:** 2023-08-04

**Authors:** Shawbo Muhamd Ahmad, Shilan Nawzad Dawood, Gollshang Ahmad Mhammed Dalloo, Tara Rasool Hussein AL-Barazanchi

**Affiliations:** Department of Conservative Dentistry, College of Dentistry, University of Sulaimani, Sulaimaniyah, 0046 Iraq

**Keywords:** Peek, E-max ceramic, Mechanical properties, Debonding, Fracture strength

## Abstract

**Background:**

Survival of endodontically treated teeth depends on an efficient restoration of the missing tooth structure.

**Objectives:**

This study aimed to investigate the mechanical properties of different endodontic post systems.

**Materials and methods:**

Human permanent maxillary central incisors (no.=58) were decoronated and root-filled. The specimens with prepared root canals were randomly divided into Group P: Polyether Ether Ketone (PEEK) post and composite core and Group C: custom-made post-core of PEEK. The cementation of the posts was carried out using self-adhesive resin cement. Nano-hybrid composite resin was used for core fabrication. After cementation, the specimens from each group were randomly divided into two subgroups according to the types of tests utilized: 14 from each group were for the fracture strength test, which was restored with IPS e-max CAD crown, and 15 from each group for the pullout resistance test. A universal testing machine was used for the test performance.

**Results:**

The fracture resistance test showed that the values significantly differed among tested posts (P = 0.013). Group P showed the highest fracture resistance. Group C exhibited higher mean pullout resistance values than the other group (P) (P = 0.059). In the two-piece PEEK post and composite core, the predominant type of failure was a core fracture, while in the one-piece PEEK post-core, most types of failures were either in the crown or in the post.

**Conclusions:**

The prefabricated posts are more resistant to fracture than the custom-made posts, with fracture and displacement mainly of the core. In contrast, both showed similar pullout resistance.

## Introduction

Efficient restoration of endodontically preserved teeth has a significant influence on the existence of the tooth. The lasting coronal tooth construction and the esthetic necessity find the restorative material and determine that the tooth is restored straightly or protected by post with core/crown [1, 2]. Cast posts have been utilized for many years owing to the fact that they follow the shape of the root cavity most sufficiently [3].

With greater demands for heightened responsiveness of aesthetics, the tooth-coloured metal-free post-core modality has become more prevalent in reinstating pulpless teeth [4]. Based on the literature, a post-like fibre post with a moderately small elastic modulus permits additional even spreading of the occlusal capacity on root dentin and cement interfaces [5]. Furthermore, utilizing tooth-coloured post substance with elastic modulus fewer than dentin like Polyether Ether Ketone (PEEK) is the crucial advantage that distributes the stress in dentin more uniformly. It assists this substance in working as a stress breaker, reducing the tooth’s catastrophic failure under excessive load [6].

Polyether Ether Ketone is a high-performance polymer that considers the latest invention of dental science and is demanded to have improved assets similar to present materials [7, 8]. In addition, PEEK can bond to the resin cement and dentin [9], making it an appropriate substance to work as a post, mainly since the maintenance of the post is accomplished by adhesive luting cement rather than the thread as the latter presents substantial stresses within the dentin [10].

Three-dimensional (3D) printed posts are the type of posts that uses 3D printing technologies which are advanced manufacturing technologies based on computer-aided design digital models to create personalized 3D objects automatically [11].

Before application of post and core, a proper endodontic treatment to the tooth is necessary to ensure adequate cleaning and shaping without causing significant harms to the root canal system [12]. There is an assumption that instrumentation of root canal system may cause micro-crack formation in the root canal walls that may influence the success of the coronal restoration. However, the use of NiTi rotary files for canal of teeth showed to reduce the risk of new micro-crack formation after instrumentation [13].

Successful restoration of endodontically treated teeth should restore the tooth’s function, esthetics and structural integrity. Therefore, direct resin composite is a widely used restorative material [14]. The two main constituents of resin composite materials are resin matrix and inorganic fillers. The resin matrix primarily consists of different types of monomers, activators and photoinitiators [15]. On the other hand, inorganic filler particles are incorporated into the resin matrix with a wide variety in size, shape, types and ratio [16]. Also, coupling agents bond fillers and resin matrix [17–19]. Dental literature showed improvement in resin matrix mainly in polymerization shrinkage, water-sorption, solubility and strength [15]. Also, crucial advancement in inorganic fillers includes the production of nano-sized filler particles and higher filler loading into the resin matrix compared to the alternatives [20, 21]. This advancement in resin composite materials improved their physical, chemical, tribological and biological properties [14, 22, 23]. Consequently, resin composite materials have been widely used not only for restoring a part of anterior and posterior teeth but also as a core material of endodontically treated teeth [24], where the core material might be subjected to high occlusal load.

Therefore, PEEK has been introduced to endodontics with potential applications as endocrown, one-piece post, core, and endodontic post with the composite body [25, 26]. However, there needs to be more information on how PEEK material behaves when used with different types of post system, core and crown materials [27, 28]. Thus, this study aimed to find the fracture resistance, mode of failure, and pullout resistance between custom-made post-core and prefabricated post-PEEK with a composite core.

## Materials and methods

### Sample selection and storage

A pilot study was conducted for each test to determine the sample size, and a power analysis was calculated using G Power 1.3.7.9 software [29]. Consequently, using T-test in both groups, significant differences were considered with an effect size of 1.23, power of 95% (a = 0.05), and samples of 14 specimens per subgroup (fracture test), while for the pullout test, an effect size of 1.38, power of 95% (a = 0.05), and samples of 15 specimens per subgroup were considered. Thus, 58 human permanent maxillary central incisors were taken from different Governmental hospitals and health centres with periodontal and maxillofacial surgery departments, and most of the departments own tooth banks. The central incisors were selected using periapical radiographs and visual inspection under a stereomicroscope at 20× magnification. The criteria for sample selection were single straight rooted teeth, no visible root caries, restorations, previous endodontic treatments, fully formed roots with mature apices and no sign of abnormal defects under a stereomicroscope (40× magnification) such as fracture, cracked tooth, external or internal root resorption. Additionally, a digital calliper measured each specimen at the cement-enamel junction to ensure a labio-palatal dimension of 5.5–6.5 mm and mesiodistal width of 6.5–7.5 mm.

### Sample preparation

The used teeth have been extracted either for periodontal, severely fractured, decayed, or un-restorable crowns. All teeth were cleaned from soft tissues and calculus and stored in 0.1% thymol solution at room temperature for one month. The teeth’ crown portions were decoronated perpendicular to their long axis to provide roots with a standard 15 mm length.

### Simulation of the periodontal ligaments and embedding of the specimens

A 0.2 mm thick aluminium foil was placed on the root and evenly adapted to the entire area. Samples were marked 2.0 mm beneath the cut surface with an elastic band to ensure that the 2.0 mm pieces of the teeth were not submerged in acrylic as described [30]. Then, a cold cure acrylic resin was introduced into the container and allowed to cure in a water bath at room temperature. Next, the specimens were removed from the acrylic blocks, the foil was removed, and a suitable amount of auto-mix light body addition silicone impression material (Empress XT, 3 M ESPE) was delivered with the dispenser gun through the mixing tip into the acrylic resin block. Finally, the tooth specimen was reseated inside the acrylic block.

### Instrumentation and obturation

Root canal instrumentation was performed with a crown-down technique using F1, F2, and F4 ProTaper Universal files (Dentsply-Sirona). In addition, 2.0 mL of 2.5% sodium hypochlorite (NaOCl) solution was used after each instrument change using 30-gauge side-vented irrigation needles. After cleaning and shaping the root canals, a final flush with 1.0 mL EDTA 17%, followed by 5.0 mL of 2.5% NaOCl to remove the smear layer. Finally, the root canals were flushed with 3.0 mL of distilled water and dried with paper points. A single-cone obturation technique with an F4 gutta-percha master cone (Maillefer-Dentsply, Ballaigues, Switzerland) and sealer (Adseal resin-based root canal sealer, Meta Biomed, Cheongju, Korea) was used to fill the root canals. The teeth were stored in an incubator at 37° C with 100% relative humidity.

### Post-space preparation and cementation

Penetration drill size two (Dentsply, Switzerland) removed the root filling up to 9.0 mm length from the sectioned surface. Next, the precision drill (Dentsply, Switzerland) size two was used in the same manner as the penetration drill to shape the post space corresponding to the selected post size 2. Then, the removal of the gutta percha was checked by radiograph.

### Sample grouping

The specimens with prepared root canals were randomly divided into two groups (29 teeth/group) according to the type of post used. Group P; Prefabricated PEEK post and composite core (two pieces), and Group C; Custom-made post-core of PEEK (one piece).

### Sample preparation

#### Group P: prefabricated PEEK post and composite core (two-piece)

PEEK posts were produced by a CAD-CAM system (Roland, DWX-50, Japan). Virtual images were obtained with the intraoral scanning system (CEREC Omnicam, Sirona, Germany) by scanning a Radix® prefabricated Fiber Post size 2 (Dentsply, Switzerland). After developing the milling plan, 29 posts were milled from the filled PEEK disc (PEEK OPTIMA®; Juvora Ltd., Wyre, Lancashire, UK). The PEEK posts were sandblasted with 50 μm Al2O3 (Masel, USA) for 15 s at a pressure of 1.5 bar, then cleaned in an ultrasonic cleaner (Guangdong, China) for 1 min; the posts were checked to ensure it’s correctly seated in the canal, then they were shortened to its final length of 12 mm with a diamond disc (Komet, Germany), finally, cleaned with alcohol and dried with water-free air. The post-cementation was done using Rely Xtm U200 Automix (3 M ESPE, USA) self-adhesive resin cement according to manufacturer instructions (Table 1). For core fabrication, nano-hybrid composite resin (Tetric Evo Ceram, Ivoclar Vivadent, Switzerland) was incrementally applied to the tooth and around the post using a plastic instrument with layers of a maximum of 2.0 mm. Each layer was polymerized by a light curing machine (Woodpecker O-Light cure unit, China) with a standard mode of 1300 MW/cm^2^ light intensity for about 40 s. The power of the light-curing units was measured with a hand-held radiometer that was recalibrated after ten times of usage. To ensure the equality of the specimens, the composite resin core build-ups were standardized using cellulose crown form of size A4 (right central incisor, crown height of 10, Dentsply, Switzerland).

**Table 1:** The compositions and working properties of the resin sealer and the resin cement used in the study

Material	Composition		Mixing Ratio	Working time (min)	Setting time (min)
	Base	Catalyst			
AD Seal resin-based root canal sealer	< 20% epoxy resin NS calcium phosphate NS zirconium dioxide NS calcium oxide NS ethylene glycol salicylate	2.5–10% N, n-dibenzyl-5-oxanonandiamin-1,9 2.5–10% amantadine	Based on volume:2 part base paste:1 part catalyst	35 at 23^0^ C	45 at 37^0^ C
Rely X^tm^ U200 Automix resin cement	Methacrylate monomers contain phosphoric acid groups, initiators, stabilizers, and rheological additives.	Methacrylate monomers, alkaline (basic) fillers, Silanated fillers, Initiator components, stabilizers, pigments, and rheological additives.	Based on volume:1 part base paste:1 part catalyst	2.30	6.0

#### Group C: custom-made post-core of PEEK (one piece)

A plastic post (PinJet, Angelus) was placed into the canal to obtain negatives of the post cavities by injecting auto-mix light body addition silicone impression material (Empress XT, 3 M ESPE) into the root canal space. The impression was completed with the heavy-body material placed in a cellulose crown form of A4 (Dentsply, Switzerland). The exact size used for group P. Digital images of the impression were obtained with an intraoral scanner (Digital photos of the impression were obtained with an intraoral scanner CEREC Omnicam, Sirona, Germany). The post-and-core was modelled with design software (Inlab CAM SW18.0 software 2.0, Dentsply Sirona, Germany). A 2.5% volume reduction in the mesiodistal and buccolingual axes was defined to allow room for the cement layer. After developing the milling plan, 29 posts were milled from PEEK discs like the previous group. The post-cementation was carried out using Rely Xtm U200 Automix (3 M ESPE, USA) self-adhesive resin cement according to manufacture instructions as in the previous group.

The specimens from each group with cemented posts were randomly divided into two subgroups according to the types of tests utilized. Twenty-eight samples (14 from each group) for the fracture strength test (P1, C1) and 30 samples (15 from each group) for the pullout resistance test (P2, C2). The crown preparation steps were only made for the 28 samples (14 from each group) in the fracture strength test.

### Crown preparation

**Fig. 1:**
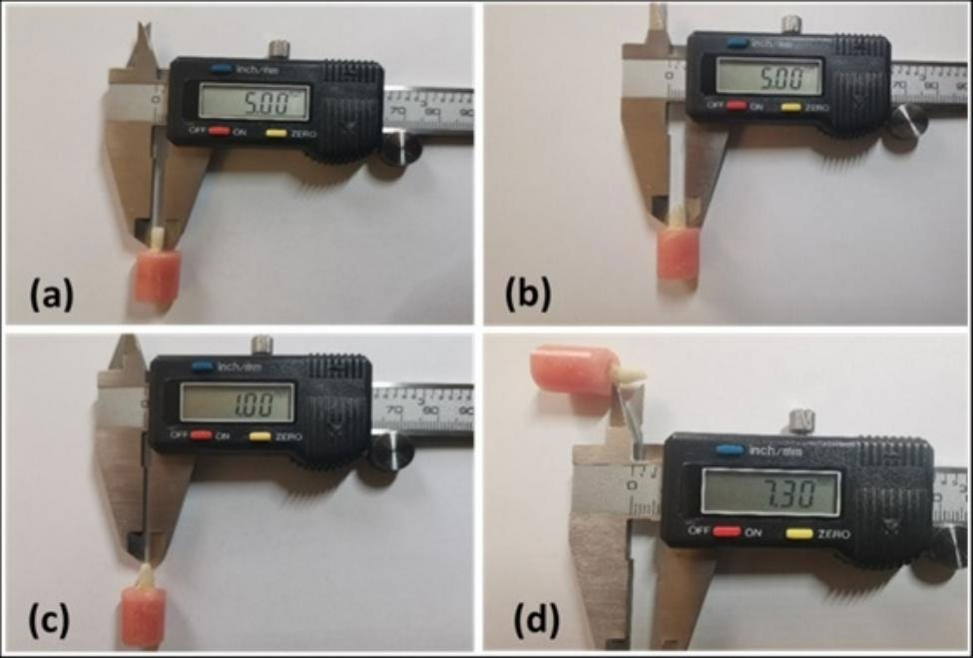
The standardized dimensions of the composite cores in all tooth samples. Mesiodistal dimension (**a**); buccolingual dimension (**b**); buccolingual dimension at incisal tip (**c**); and height of the core (**d**)

Each sample was prepared to receive an all-ceramic crown according to the Fundamentals of fixed prosthodontics [31], using (6847KR.31.016 FG modified flat end tapered diamond bur, 6379-023 football-shaped diamond bur) in a high-speed handpiece under the cold-water spray. The preparation’s finish line was placed 2.0 mm apical to the core/tooth junction, leaving a 1.0 mm wide radial shoulder on the facial and 0.8 mm wide in other areas. The wall convergence had approximately 6º and was standardized by fixing the bur vertically by the dental surveyor during finish line preparation. Ultimately, all the practices yielded similar dimensions (measured with a digital calliper) mesiodistally and faciolingually (Fig. 1).

### Crown fabrication

**Fig. 2:**
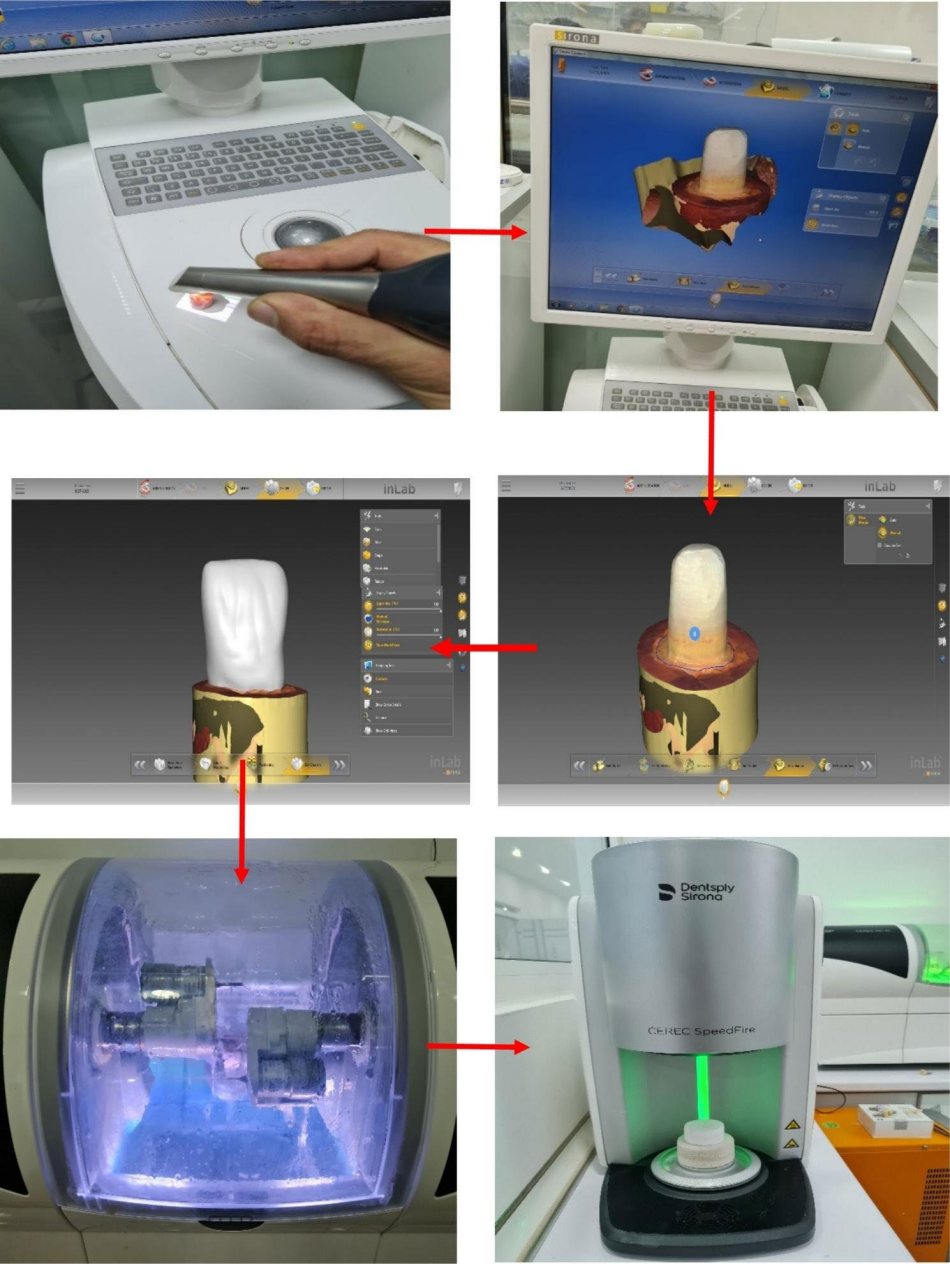
The process of crown formation. The red arrows showing the order in the steps of crown formation

The crowns were fabricated with particular attention to maintaining the standardization of ceramic thickness for samples (Fig. 2). The crowns were completed in four phases. In the administration phase, the crown was selected as the restoration type from single restoration options. The maxillary central incisor tooth was chosen as the abutment tooth, while in the scan phase, the prepared teeth were digitally scanned using CEREC Omnicam. The crown was then designed through the model phase by transferring the data to Inlab CAM SW18.0 software for precise process and creating the crowns into standardized sample sizes. The system automatically detected the margin of preparation. Finally, in the design phase, the crowns were milled from E-max blocks in a milling machine into a pre-crystallized state, the restoration was separated from the spindle of the engine, and the sprue was cut using a fine diamond disk. Next, the sprue attached area was smoothed using a polishing disk, and the restorations were cleaned thoroughly under running water and dried; then, they were placed on the honeycomb tray to be crystallized. Finally, the crowns were fully crystallized in a special furnace automatically programmed by a manufacturer specialized in the used material.

### Crown cementation

The received crown restoration was inspected and checked for fitness and adaptation. Then, all the prepared samples were rinsed with water spray and lightly air-dried. Next, the inner surfaces of the crowns were etched with 5% hydrofluoric acid for 20 s; then, they were rinsed off and dried. After that, Prime & Bond® adhesive (Dentsply, Switzerland) was applied onto the pretreated crown surface for salinization. Subsequently, the crown was filled with the Rely Xtm U200 Automix self-adhesive resin cement; then, the height was seated firmly on the core. After 3 min, the excess glue was removed by a sharp explorer. Finally, all cemented specimens were stored in distilled water at 37° C in an incubator for 24 h before being subjected to thermocycling (ISO/TS 11,405, 2003).

### Fracture resistance test

**Fig. 3:**
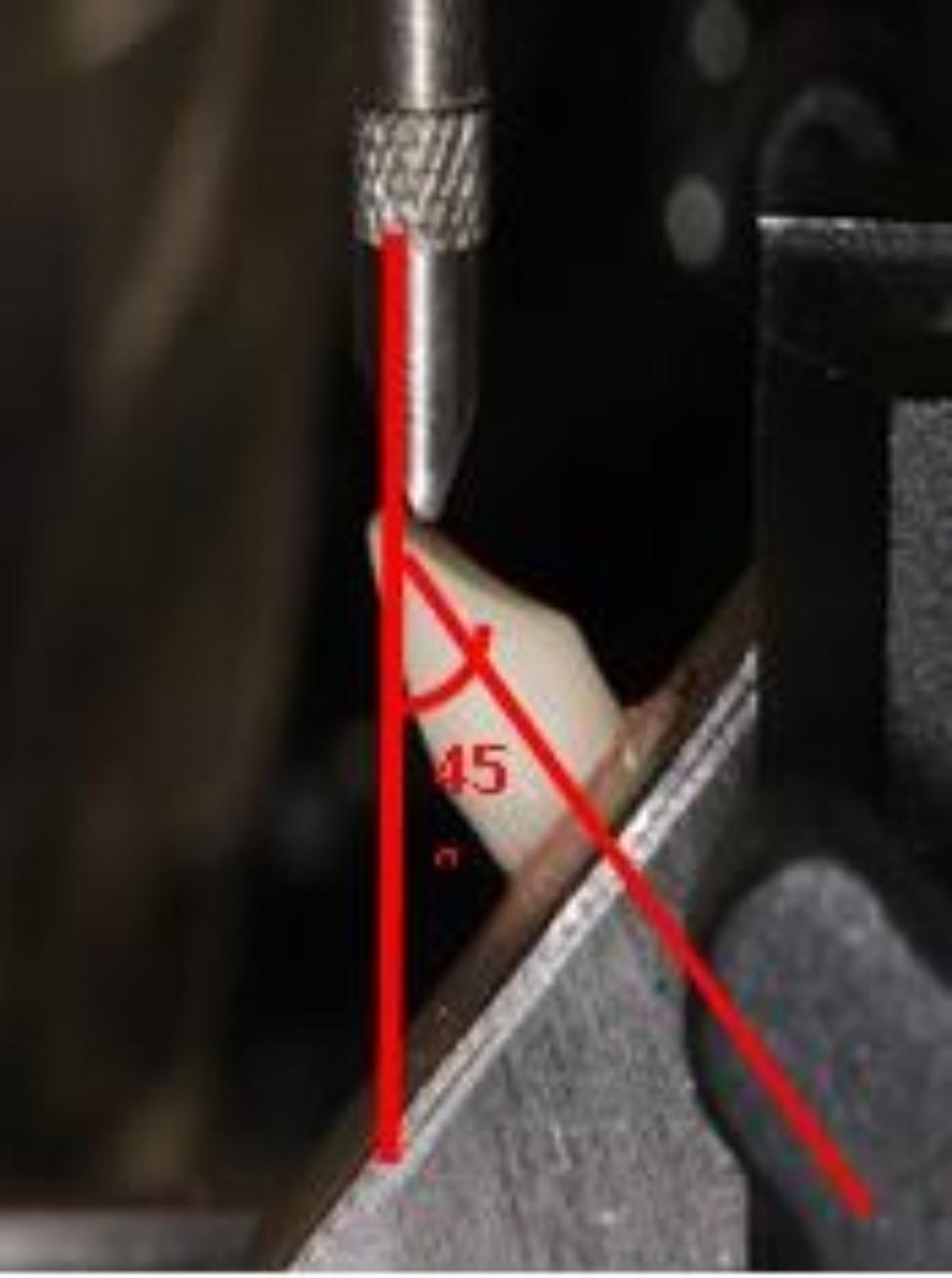
Fracture test of the samples under the universal testing machine. The blade applied 3.0 mm apical to the incisal margin of the palatal surface of the crown at an angle of 45^0^ to the longitudinal axis of the tooth

Each specimen was placed in a custom apparatus fixed to the lower arm of the universal testing machine (Lloyd LRX, Lloyd Instruments Fareham, England) (Fig. 3) that allowed the specimens to be positioned at 45° to their long axis to simulate the loading conditions applied in vivo. A metal indenter with a 6.0 mm width and 1.0 mm thickness was fixed to the upper arm of a universal testing machine. A static site loaded with a crosshead speed of 1.0 mm/min was applied 3.0 mm apical to the incisal margin of the palatal surface of the crown at an angle of 45^º^ relative to the longitudinal axis of the tooth for calculating stress distribution in regular occlusal contact with the antagonist’s tooth in a universal testing machine until failure. The loads were measured in Newton (N). The mode of failure was recorded and classified from less catastrophic fracture (displacement of crown, fracture of the crown, and fracture of the core) to more catastrophic fracture (fracture of the post and fracture of the tooth), which was challenged to restore [26, 32].

### Pullout resistance tests

The remaining 15 samples from each group (30 pieces) were placed and fixed tightly in custom apparatus to prevent movement during testing. The custom apparatus was selected for the lower arm of the universal testing machine that allowed the posts to be positioned vertically. In contrast, the core was fitted to dynamometer clamps of the upper arm of the device. The pullout test was performed parallel to the long axis of the post at a crosshead speed of 1.0 mm/min in a universal testing machine. The maximum force required to dislodge each post was recorded in Newton (N).

### Statistical analysis

Statistical analyses were made using standard software (IBM SPSS; version 25). All data were submitted to the Shapiro–Wilk test to verify the normality of data. Descriptive results were shown as means and standard deviation (SD) for quantitative data and as frequency (%) for categorical data. T-test was used to compare data of pullout and fracture strength tests between groups, while the Chi-square test was used to compare failure rate modes. *P*-values < 0.05 were considered to be statistically significant in all tests.

## Results

The results of the pullout and fracture strength tests were not statistically significant (p > 0.05) using the Shapiro–Wilk test, meaning that the data does not differ significantly from the normally distributed. However, the mean values of fracture resistance were significantly different among tested posts (p = 0.013), with the highest mean fracture resistance (416 ± 49 N) of Group P1 (Table 2).

**Table 2:** Comparison of the mean fracture resistance between the tested groups

Groups	Mean fracture resistance	No.	P-value
P1	416.14 ± 49.35	14	0.013*
C1	365.5 ± 62.26	14	

*: Significant difference using t-test, C1: Custom-made post-core of PEEK; P1: Prefabricated PEEK post and composite core

**Fig. 4:**
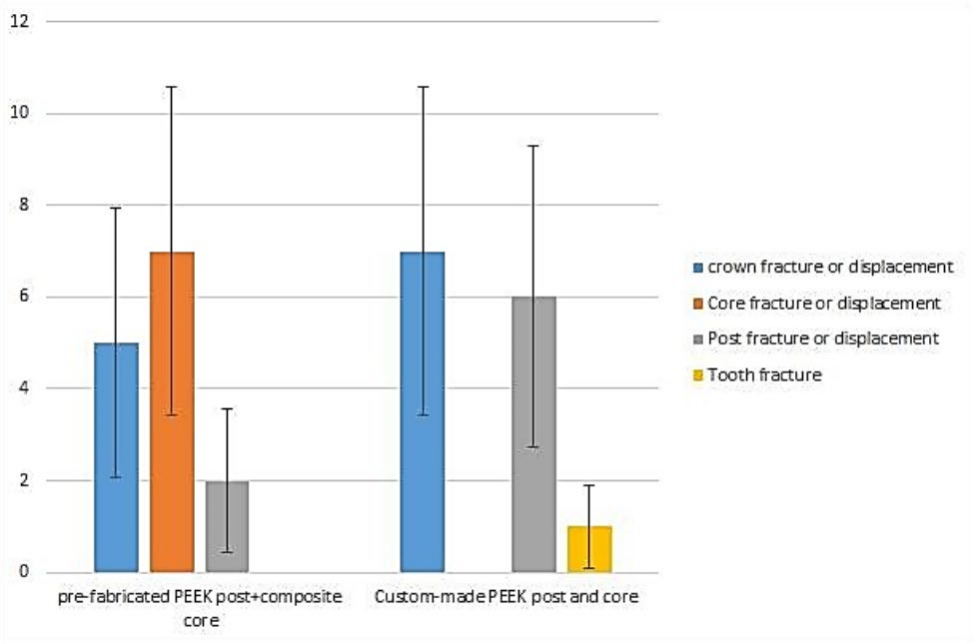
The bar chart chows the distribution of the fracture type among the tested groups. The bars displayed standard errors

**Fig. 5:**
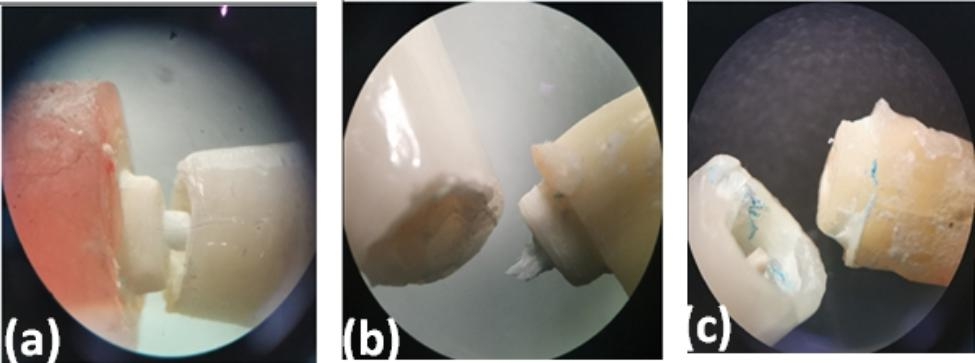
Types of fracture modes under stereomicroscope. Core fracture (**a**); crown fracture (**b**); and post fracture (**c**)

There was a significant difference in the failure modes using the Pearson Chi-square test (p = 0.16) between the studied groups (Fig. 4). In the two-piece PEEK post and composite core, the predominant type of failure was core displacement or fracture (Fig. 5a), while in the one-piece PEEK post-core, most types of losses were either in the crown or in the post (Fig. 5b and c). Although the custom-made one-piece post and core PEEK post group (C2) exhibited higher mean values of pullout resistance compared with the other group (P2), the difference was insignificant (p = 0.059) (Table 3).

**Table 3:** Comparison of the mean pullout resistance between the tested groups

Groups	Mean Pull-out resistance	No.	P-value
P2	573.07 ± 84.53	15	0.059*
C2	599.73 ± 119.20	15	

*: Significant difference using t-test, C2: Custom-made post-core of PEEK; P2: Prefabricated PEEK post and composite core

## Discussion

The risk of tooth fracture is an undesirable incident usually related to insufficient coronal tooth structure after endodontic treatment [33]. Rebuilding the tooth structure by a post before crown restoration is sometimes mandatory to provide stable and solid tooth restoration [34]. Due to the more precise control of biomechanical parameters and absence of uncontrolled variables inherent to clinical trials, the combined results and conclusions of the most relevant in vitro studies regarding the basic recommendations for material selection and treatment of pulpless teeth lead to highlight the composition and structural alterations resulting from the loss of pulp vitality and endodontic and various restorative procedures [35]. Therefore, in the present study, an in vitro assessment was adopted to evaluate the fracture and pullout resistance of a PEEK prefabricated post with a custom-made post and core as a conservative alternative approach that aims to restore endodontically treated teeth.

It has been observed that fracture resistance of restoration with a post is directly related to post design, post length, post diameter, core material, and type of cement used [2]. Root fracture occurs when this stress transmission exceeds the withstanding resistance of the treated tooth against masticatory forces [36]. In earlier days, the rationale for using stiffer posts has always been to strengthen the tooth. However, this concept is questioned because of the existing advancement in adhesive procedures; a post with biomechanical properties compatible with dentin acts as a monoblock system against root fracture [35] because the root fracture is considered one of the most frustrating complications in endodontically treated teeth which account as a severe clinical concern with an unfavourable prognosis [37].

Most in vitro studies of teeth restored with post and core restorations used the fracture resistance test, as it is considered the main recommended characteristic to achieve durable restoration [38]. To reproduce the natural condition in the oral cavity, a thin layer of silicone rubber base impression simulating periodontal ligaments and acrylic resin blocks were used to simulate the bone. Since rigid reinforcement of the root may alter the strengths of the roots, along with the patterns of failure [39]. In addition, thermal cycling was applied to simulate the thermal degradation of the posts and the dentin with the resin cement under clinical conditions. It was proven that thermal cycling influenced the bond strengths of self-adhesive resin cement to fibre posts [40].

The crown placement has been questioned as this might obscure the effects of a different post [41]. However, testing after post and core placement without a crown would not reflect everyday clinical practice. Therefore, all samples in this study were restored and tested with ceramic crown coverage for the fracture test. It was noted that the prefabricated PEEK post with the composite core groups (P1) had a higher resistance to fracture when compared to the one-piece PEEK post and core (C1). The previous studies compared the fracture test evaluation of several types of post materials with PEEK post or the analysis between heat-pressed and CAD-CAM fabrication methods; no article studied the design of the PEEK endodontic post. This result may be owed to the flexion of the one-piece PEEK post and core under functional stress, which may result in micromovement of the core, producing documentation of the crown or post fracture, while in the composite core group; the composite has higher elastic modulus than PEEK material [25], which may act as a supporting structure to the ceramic crown. Moreover, Teixeira et al., 2020 compared fracture resistance of custom-made Post-and-cores of PEEK material and concluded that customized post-and-cores of PEEK exhibited good mechanical performance when compared to the conventional cast post materials [26].

In addition, in the present study, the groups restored with PEEK post and composite core exhibited less catastrophic fracture, which usually renders the tooth non-restorable and requires extraction. Generally, in both groups, the number of repairable fractures was predominant. The present study’s results are consistent with previous studies [26, 42].

The biocompatible chemical composition and the low surface energy of PEEK materials may lead to difficulties bonding to resin-based materials [43]. However, mechanical and chemical surface pretreatments effectively promoted bonding among PEEK, cement and dentin. Moreover, the effect of different adhesive systems as subsequent pretreatment and conditioning steps is of considerable interest in improving adhesion [44]. Thus, in this study, custom-made one-piece PEEK post produced a higher bond strength when compared to other studied groups; However, the result was not significant; it supports the idea that good post adaptation increases frictional retention, resulting in better performance of the post-core and crown systems [45]. This result is consistent with the findings of another study, which concluded that the bonding properties of PEEK posts are suitable alternative post systems for clinical application [46].

This in vitro study had several limitations, making comparing the results with clinical situations difficult. The first limitation concerns that the fatigue test is necessary to determine the long-term survivor of an endodontically treated tooth restored with a post. Another difficulty was encountered in controlling related factors clinically, such as when extracted teeth were used for this study, the potential for significant uncontrollable variations in strength exists, for example, the age of the teeth used in the fracture test, as most of the teeth were extracted in old persons for the periodontal cause. It’s known that the changes in the microstructure of dentin with age cause a degradation in the fatigue and fracture properties [47].

## Conclusion

Prefabricated PEEK posts with composite core had better performance about fracture resistance and failure modes compared to the PEEK post-core, while they exhibited similar behaviour against pullout resistance. The results of this study can be considered of clinical significance because the findings outline the potential scope of the proposed material in improving the outcomes obtained with a prefabricated endodontic post to provide a better understanding of the stress distribution with their effect on fracture resistance and debonding between the components of post endodontic restoration, which help in selecting the most appropriate combination of materials that meet the overall system objective.

## Data Availability

The datasets used and analyzed during the current study are available from the corresponding author upon reasonable request.
